# Inferior outcome after hip resurfacing arthroplasty than after conventional arthroplasty: Evidence from the Nordic Arthroplasty Register Association (NARA) database, 1995 to 2007

**DOI:** 10.3109/17453674.2010.525193

**Published:** 2010-10-08

**Authors:** Per-Erik Johanson, Anne Marie Fenstad, Ove Furnes, Göran Garellick, Leif I Havelin, Sören Overgaard, Alma B Pedersen, Johan Kärrholm

**Affiliations:** ^1^The Swedish Hip Arthroplasty Register, Department of Orthopedics, Institute of Surgical Sciences, Sahlgrenska University Hospital, University of Gothenburg, Sweden; ^2^The Norwegian Arthroplasty Register, Department of Orthopedic Surgery, Haukeland University Hospital, Bergen; ^3^Department of Surgical Sciences, University of Bergen, Norway; ^4^The Danish Hip Arthroplasty Register and Department of Orthopedic Surgery, Clinical Institute, Odense University Hospital, Odense; ^5^The Danish Hip Arthroplasty Register and Department of Clinical Epidemiology, Aarhus University Hospital, Aarhus, Denmark

## Abstract

**Background and purpose:**

The reported outcomes of hip resurfacing arthroplasty (HRA) vary. The frequency of this procedure in Denmark, Norway, and Sweden is low. We therefore determined the outcome of HRA in the NARA database, which is common to all 3 countries, and compared it to the outcome of conventional total hip arthroplasty (THA).

**Methods:**

The risk of non-septic revision within 2 years was analyzed in 1,638 HRAs and compared to that for 172,554 conventional total hip arthroplasties (THAs), using Cox regression models. We calculated relative risk (RR) of revision and 95% confidence interval.

**Results:**

HRA had an almost 3-fold increased revision risk compared to THA (RR = 2.7, 95% CI: 1.9–3.7). The difference was even greater when HRA was compared to the THA subgroup of cemented THAs (RR = 3.8, CI: 2.7–5.3). For men below 50 years of age, this difference was less pronounced (HRA vs. THA: RR = 1.9, CI: 1.0–3.9; HRA vs. cemented THA: RR = 2.4, CI: 1.1–5.3), but it was even more pronounced in women of the same age group (HRA vs. THA: RR = 4.7, CI: 2.6–8.5; HRA vs. cemented THA: RR = 7.4, CI: 3.7–15). Within the HRA group, risk of non-septic revision was reduced in hospitals performing ≥ 70 HRAs annually (RR = 0.3, CI: 0.1–0.7) and with use of Birmingham hip resurfacing (BHR) rather than the other designs as a group (RR = 0.3, CI: 0.1–0.7). Risk of early revision was also reduced in males (RR = 0.5, CI: 0.2–0.9). The femoral head diameter alone had no statistically significant influence on the early revision rate, but it eliminated the significance of male sex in a combined analysis.

**Interpretation:**

In general, our results do not support continued use of hip resurfacing arthroplasty. Men had a lower early revision rate, which was still higher than observed for all-cemented hips. Further follow-up is necessary to determine whether HRA might be useful as an alternative in males.

The development of contemporary metal-on-metal (MOM) bearings has stimulated renewed interest in hip resurfacing arthroplasty (HRA) of the hip. These devices are available in different designs, most of which are hybrid concepts with cemented femoral and uncemented acetabular components. The proposed advantages of HRA compared to conventional total hip arthroplasty (THA) include improved range of motion and hip function, bone preservation, lower dislocation rates, and easier and safer revision procedures in case of failure (Shimmin et al. 2008). Because of the low wear characteristics observed in the laboratory and in clinical situations, the MOM bearing is thought to be especially suitable for patients with a long life expectancy (McMinn and Daniel 2006).

Early reports from specialized centers have shown high survival rates: 97.8–99.8% after 3–5 years (Daniel et al. 2004, Treacy et al. 2005, Hing et al. 2007). Other authors have reported inferior results (Kim et al. 2008, Stulberg et al. 2008). Narrowing of patient selection criteria and refinement of surgical technique have improved the results in some case series (Mont et al. 2007, Amstutz et al. 2007). Several studies have shown that HRA is associated with a long learning curve. Early failures or inadequate implant positioning occurred at the beginning of the learner's case series, which tended to decrease thereafter (Marker et al. 2007, Witjes et al. 2009, Nunley et al. 2010). Early failures are most commonly caused by femoral neck fracture and aseptic loosening of the femoral component. Thus, there are several indications that the outcome of HRA is influenced by patient selection, surgical technique, and experience in using this type of implant.

Short- and medium-term results of HRA have previously been reported from national joint replacement registries. There has been a rapid increase in the use of HRA, with varying percentages of HRA relative to the total volume of THAs reported by different registries (Kärrholm et al. 2008, CJRR 2008-2009, AOANJRR 2008). Reports of inferior results, except in younger males with primary osteoarthritis, are most probably responsible for the recent tendency of decreasing use of HRA—especially in females (AOANJRR 2008). Further reports of comparatively rare but serious complications (Pandit et al. 2008, Hart et al. 2009, Ollivere et al. 2009) have probably also contributed to this tendency. Poor results after revision of failed HRA, equal to those obtained after revision of THA (AOANJRR 2008), may also have contributed to more restricted use.

We analyzed the early outcome concerning aseptic revisions within 2 years of HRA and compared it to that of THA in the common database of the Nordic Arthroplasty Register Association (Havelin et al. 2009). We also evaluated the extent to which outcome was influenced by implant design, number of procedures per hospital, and femoral head size.

## Patients and methods

### Inclusion criteria

The NARA database consists of data on individual total hip replacements compiled from the 3 national joint replacement registry databases of Denmark, Norway, and Sweden (Havelin et al. 2009). At the time of this analysis, the database covered 309,290 operations performed between 1995 and 2007.

To reduce the skew in demographic distribution between patients operated with HRA and those operated with THA, cases older than 73 years of age (the oldest patient operated with HRA) and those with femoral neck fracture were excluded (there was 1 case in the HRA group, probably a registration error and 34,944 in the THA group). 461 hips (0.3%) with missing information about type of implant, 4 about operated side, and one about gender were also excluded, leaving 1,638 HRAs and 172,554 THAs available for analysis (Table 1). Due to the short follow-up in the HRA group (mean 1.8 years (SD 1.5); THA: 5.1 (SD 3.5)) revision within 2 years was used as endpoint. Only aseptic revisions were included. Revision was defined as removal or exchange of at least one of the components.

**Table 1.** The total study group of 174,192 THA patients up to 73 years of age (those with femoral fractures excluded) who were operated 1995 through 2007

	HRA	THA	p-value
	n = 1,638	n = 172,554	
Percentage males	68	43	< 0.001 **^a^**
Mean age, years (range)	51 (15–73)	62 (12–73)	< 0.001 **^b^**
Age groups, %			
< 30	2.1	0.6	
30–39	8.9	1.8	
40–49	31	6.1	
50–59	42	23	
60–74	17	69	< 0.001 **^a^**
Operated side, % (no.)			
Right	53 (861)	54 (93,866)	0.1 **^a^**
Diagnosis, %			
Primary osteoarthritis	89	85	
Inflammatory arthritis	2.2	4.3	
Childhood diseases	6.5	6.1	
Idiopathic femoral head necrosis	0.9	2.7	
Other	1.0	2.1	< 0.001 **^a^**

**^a^** Chi-squared test.

**^b^** Non-parametric.

### Statistics

Kaplan-Meier survival analysis was used to estimate the unadjusted cumulative revision rates, presented as cumulative survival (CS) with 95% confidence interval (CI). Adjusted revision rates were calculated using Cox multiple regression analysis. The proportional hazards assumption was controlled for by performing hazard function and log-minus-log plots for strata of each covariate separately. Plots were investigated for acceptable proportionality between hazard functions of the different covariate strata and for strictly parallel log-minus-log plots. No crossing or clearly deviating lines were accepted. No continuous variables were entered in the model.

Inclusion of bilateral cases in a survival analysis violates the basic assumption that all cases analyzed are independent. However, several reports have shown that the effect of including bilateral cases in studies of hip and knee joint prosthesis survival is negligible (Schwarzer et al 2001, Robertsson and Ranstam 2003, Lie et al 2004). We therefore included all available cases to maximize statistical power. Relative risk (RR) estimates were calculated and presented with 95% CI. The level of significance was 95%. We used the SPSS statistical software package version 16.0.

### Hip resurfacing vs. conventional implant designs

HRA was compared to all-cemented THA and THA as a group (all-cemented, non-cemented, hybrid, and inverse hybrids) with adjustment for age at surgery, sex, operated side, diagnosis, and nationality, using Cox multiple regression. Age was classified as 0–49 years, 50–59 years, or ≥ 60 years. Diagnosis was entered as primary or secondary osteoarthritis, the latter including non-fracture diagnoses other than primary osteoarthritis. In addition, stratified analyses were done for males and females aged 49 years and younger, and between 50–73 years of age, dividing the HRA group by its mean age.

### Hip resurfacing group

The HRA group was further analyzed with regard to the influence of age at surgery (classified as 0–49 years or ≥ 50 years), sex, diagnosis, implant design, and number of HRAs performed per hospital (2 groups: total numbers < 70 or ≥ 70) on risk of revision within 2 years. The limit of 70 patients was set arbitrarily based on the actual distribution of cases per hospital, and also so that each country contributed with at least one high-volume hospital. Operated side was not included in the analysis since this covariate did not fulfill the proportional hazards assumption in this group, showing an increase in revision rate for left-sided hips at the end of the 2-year period. The 4 most commonly used implant designs were studied (ASR, DePuy; BHR, Smith and Nephew; Durom, Zimmer; ReCap, Biomet) (Table 2), resulting in a study group of 1,611 hips with 35 revisions. The remaining 27 hips of other designs were excluded from this analysis because of a clearly deviating survival function, thus not fulfilling the proportional hazards assumption necessary for the Cox regression model. We did not adjust for nation in the analysis of HRA designs because all designs of HRA were not used in all 3 countries. Information about femoral head diameter was available in 1,552 cases. To reduce the influence of missing observations and to investigate correlation with sex, femoral head diameter (classified as ≤ 44 mm, 45–49 mm, 50–54 mm, and ≥ 55 mm) was added in separate evaluations. This is the same diameter classification as in the Australian Registry report (AOANJRR 2008).

**Table 2.** HRA implant designs in the NARA database, 1995–2007

Implant design	%	n
BHR	48	780
Durom	21	344
ASR	18	296
ReCap	12	191
Adept	0.9	14
Cormet (+/- HA)	0.4	7
McMinn	0.4	6
Total		1,638

### Ethics

The study was approved by the Danish Data Protection Agency (J. no. 2008-41-2024), the Norwegian Social Science Data Services, and the Swedish Data Inspection Board.

## Results

### Demographics and revisions

68% of patients were male in the HRA group and 43% were male in the THA group. Mean age of HRA cases was 51 (15–73) years and mean age of THA cases was 62 (12–73) years. Primary osteoarthritis was slightly more common in the HRA group (89%) than in the THA group (85%) (Table 1). 107 of the 174 cases with secondary arthritis in the HRA group were classified as secondary to childhood diseases. The HRA designs in the database are listed in Table 2. The 1-year unadjusted Kaplan-Meier-estimated cumulative revision rate was 1.8% for HRA and 0.7% for THA, and the 2-year rates were 3.3% and 1.2% (Table 3). The main reason for early revision of hip resurfacings was fracture, whereas THAs were revised early mainly because of dislocation. Unspecified reasons for revision (“other”) were recorded in 27% of the HRA revisions as compared to 9% in THA revisions, the high numbers in the former group being due to the limitations of detailed revision data that were accessible.

**Table 3.** Unadjusted revision rates and reasons for aseptic revision up to 2 years

	HRA	THA	p-value
	n=1,638	n=172,554	
Crude aseptic revision rate, % (no.)			
1 year	1.6 (26)	0.7 (1251)	
2 years	2.4 (40)	1.1 (1954)	
Kaplan-Meier unadjusted non-septic cumulative revision rate, % (95% CI)			
1 year	1.8 (1.1–2.4)	0.8 (0.7–0.8)	
2 years	3.3 (2.2–4.3)	1.2 (1.2–1.3)	< 0.001 **^a^**
Reason for revision up to 2 years, % (no.)			
Aseptic loosening	25 (10)	25 (497)	
Fracture	40 (16)	9 (176)	
Dislocation	0	50 (967)	
Pain only	5 (2)	4 (88)	
Other	30 (12)	12 (226)	< 0.001 **^b^**

**^a^** Log-rank test.

**^b^** Chi-squared test.

### Hip resurfacing vs. conventional implant designs—overall results

**Figure 1.**
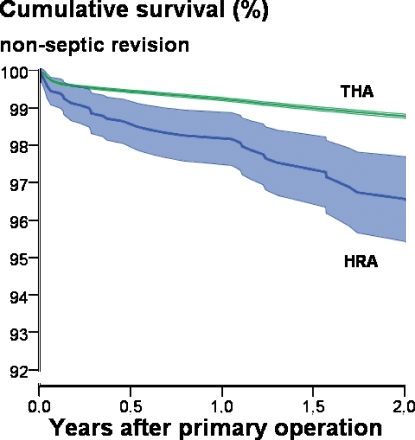
Unadjusted Kaplan-Meier-estimated cumulative survival (CS ± 1.96 SE) for 1,638 HRAs with estimated 2-year CS of 96.7% (95% CI: 95.7–97.8; no. remaining = 547) and 172,554 THAs with estimated 2-year CS of 98.8% (95% CI: 98.7–98.8; no. remaining = 135,173); p < 0.001 (log-rank test).

Figure 1 shows unadjusted Kaplan-Meier-estimated cumulative survival for the HRAs and for the total group of THA implants. In the Cox regression analysis, HRA showed an increased risk of early aseptic revision compared to THA (RR = 2.7, CI: 1.9–3.7; p < 0.001) and all-cemented THA (RR = 3.8, CI: 2.7–5.3; p < 0.001).

### Hip resurfacing vs. conventional implant designs—age and sex analysis

In the older age group of men, 50–73 years, the early revision rate of HRA was increased compared to all cemented THAs (RR = 2.1, CI: 1.1–3.9), but not when compared to the compound group of all variations of THAs. Conversely, females between 50 and 73 years of age with HRA had an increased risk of early revision both compared to THA (RR = 3.2, CI: 1.6–6.5) and to all-cemented THA (RR = 4.0, CI: 2.0–8.1). In the younger subset of males (≤ 49 years), the early revision rate of HRA was again increased compared to all-cemented THA (RR = 2.4, CI: 1.1–5.3), but not when compared to THA as a whole. In younger females, the risk of early revision of HRA implants was 4.7 (CI: 2.6–8.5) compared to THA as a whole and 7.4 (CI: 3.7–15) compared to all-cemented THA (Table 4).

**Table 4.** Age- and sex-stratified relative risk of aseptic revision within 2 years after primary operation. HRA was compared to all other types of conventional THA or only to the all-cemented (all-c THA) subpopulation. Data are based on a Cox regression model adjusted for operated side, nationality, diagnosis, and type of implant

	Adjusted revision risk (95% CI)				Number of cases (No. of revisions)		
	HRA/THA	p-value	HRA/all-c THA	p-value	HRA	THA	all-c THA
Age < 50 years							
Males	1.9 (1.0–3.9)	0.07	2.4 (1.1–5.3)	0.04	460 (9)	7,183 (109)	1,782 (18)
Females	4.7 (2.6–8.5)	< 0.001	7.4 (3.7–15)	< 0.001	221 (13)	7,486 (114)	2,242 (21)
Age 50–73 years							
Males	1.7 (0.9–3.1)	0.1	2.1 (1.1–3.9)	0.02	653 (10)	67,134 (807)	43,725 (387)
Females	3.2 (1.6–6.5)	0.001	4.0 (2.0–8.1)	< 0.001	304 (8)	90,751 (924)	64,308 (482)

### Hip resurfacing group: the 4 most commonly used designs

The mean overall follow-up for ASR was 1.1 (0.0–3.1) years; for BHR it was 2.1 (0.0–8.1) years, for Durom it was 2.1 (0.0–5.8) years, and for ReCap it was 1.1 (0.0–2.8) years. Males had a 50% reduced risk of early revision (RR = 0.5, CI: 0.2–0.9) (Table 5). Repeat of the analysis including also the femoral head diameter reduced the number of observations to 1,552. In this combined analysis, neither femoral head diameter nor gender had any statistically significant influence on early revision rate (Table 6). Finally, in performing the same analysis but omitting gender, femoral head diameter alone did not have any statistically significant influence on early revision rate (data not shown). Femoral heads with diameters of 50–54 mm had lower revision rates, but the difference was not significant. Hospitals that had performed less than 70 HRA procedures had an almost 4 times increased risk of early revision (RR = 3.7; CI: 1.5–8.8) compared to those with a longer record (Tables 5 and 6).

**Table 5.** Relative risk of aseptic revision up to 2 years (RR) with 95% confidence interval (CI) in 1,611 hip resurfacings (35 revisions). Data are based on a Cox regression model including age (< 50 or ≥ 50 years), sex, diagnosis, hospital production volume, and the 4 most common HRA designs with BHR as reference

	RR	95% CI	p-value
Hospital production volume:			
< 70 / ≥ 70 procedures	3.7	1.5–8.9	0.003
Hip resurfacing design:			
BHR (reference)	1	–	0.02
Durom	3.1	1.2–7.8	0.02
ASR	2.7	1.0–7.4	0.06
ReCap	5.4	1.8–16	0.003
Male / female	0.5	0.2–0.9	0.03
Age (< 50 / 50–73 years)	1.5	0.8–3.1	0.2
Primary / secondary OA	1.0	0.4–2.8	0.9

**Table 6.** The same type of analysis as in Table 5 except that femoral head diameter (categorized as ≤ 44mm, 45–49 mm, 50–54 mm, and ≥ 55 mm) has been added, resulting in 1,552 hip resurfacings (35 revisions) available for analysis

	RR	95% CI	p-value
Hospital production volume:			
< 70 / ≥ 70 procedures	3.7	1.5–8.8	0.004
Hip resurfacing design:			
BHR (reference)	1	–	0.02
Durom	3.1	1.2–7.8	0.02
ASR	2.3	0.8–6.5	0.1
ReCap	5.4	1.8–17	0.003
Male / female	0.7	0.3–1.8	0.5
Femoral head diameter			
< 44 mm (reference)	1	–	0.3
45–49 mm	0.8	0.3–2.4	0.8
50–54 mm	0.4	0.1–1.4	0.2
> 55 mm	0.9	0.2–4.2	0.9
Age (< 50 / 50–73 years)	1.4	0.7–3.0	0.3
Primary / secondary OA	1.2	0.4–3.4	0.7

**Figure 2.**
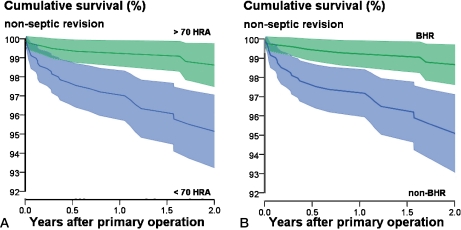
Unadjusted Kaplan-Meier-estimated cumulative survival (CS) of the group of the 4 most common types of HRA, subgroups with significant influence on early non-septic revision rate (CS ± 1.96SE). A. Hospital with ≥ 70 HRAs: n = 820, 2-year CS = 98.8% (95% CI: 97.9–99.8; no. remaining at 2 years = 269). Hospital with < 70 HRA: n = 791, 2-year CS = 95.5% (95% CI: 93.7–97.2; no. remaining at 2 years = 272); p < 0.001 (log-rank test). B. BHR: n = 780, 2-year CS = 98.8% (95% CI: 97.9–99.7; no. remaining at 2 years = 341). Non-BHR: n = 831, 2-year CS = 95.6% (95% CI: 93.8–97.3; no. remaining at 2 years = 200); p < 0.001 (log-rank test).

When we compared the different HRA designs using BHR as a reference, Durom, ASR, and ReCap all had a higher risk of early revision, even though the difference with ASR was just outside the limit of statistical significance. CI for the Durom, ASR, and ReCap designs showed considerable span and overlap, and the analysis does not permit any ranking between them. Compared to the other designs pooled into one group, BHR had a lower adjusted risk of early revision (RR = 0.3, CI: 0.1–0.7; p = 0.008). Figure 2 shows unadjusted Kaplan-Meier cumulative survival for those factors that turned out to have a statistically significant influence.

## Discussion

National joint replacement registers reflect the broad use of joint prostheses. Good results reported from highly specialized centers are often not reproduced when a new implant design is introduced to all orthopedic surgeons, especially if the procedure is technically demanding and suitable only for a small patient population. Register reports correspond to observational studies and they may suffer from different types of bias. On the other hand, they survey large numbers of patients. In the 3 Scandinavian countries included in this study the frequency of these procedures is low, which underscores the value of compilation of the national databases.

### Limitations of the study

Our results may have been influenced by confounding. In the HRA group, there was more primary osteoarthritis and less inflammatory and other types of secondary OA than in the reference group, possibly favoring prosthesis survival in the HRA cohort. Despite the use of an upper age limit, patients in the control group had a higher mean age. These problems were adjusted for as far as possible by the use of regression models. Unmeasured confounding, such as bone quality, the anatomy of the hip joint, and the preferences of patient and surgeon would certainly also influence the results, but these considerations could not be addressed in our study.

The follow-up was short. This means that the proportion of revisions that could be related to surgical and technical errors was high. With longer follow-up, other reasons for revision—and especially those related to loosening, wear, and biological reaction caused by wear-related debris or release of metal—can be supposed to change the relative distribution of revisions in either direction. Also, there were relatively few revisions, permitting only a minimum of stratified analysis and increasing the sensitivity to random effects of single revision cases.

In the total group of THA patients aged less than 74 years and with a non-fracture diagnosis, 461 hips were excluded because of missing information about type of implant. If a substantial fraction of those were HRA cases, we may have underestimated the risk of early revision after HRA. The crude revision rate in this group was 1.7% (8/461) with a mean age of 54 years, and the proportion of males was 52%, indicating a case mix closer to that of the HRA group than to that of the THA group or the combined group.

### Hip resurfacing vs. conventional implant designs

Annual reports from national joint replacement registers have shown increased revision rates of hip resurfacing compared to conventional arthroplasty (NJR England and Wales 2009, Kärrholm et al. 2008, AOANJRR 2008). We could confirm this observation but found that for men of any age, there was no statistically significant difference in early revision rate for THA as a group compared to HRA, but that there was a reduced revision rate for all-cemented THA vs HRA. In the British registry, the results of hip resurfacing are improved for males less than 55 years of age but they do not, however, match those of THA (NJR England and Wales 2009). In the Australian registry, the 3-year revision rate for HRA is the same as for THA in the group of patients less than 55 years of age, and the revision rate of HRA increases with age (Buergi and Walter 2007). Studies comparing HRA with different types of THA for corresponding age groups have shown similar results (Pollard et al. 2006, Vail et al. 2006). However, in the NARA database there is a considerable increase in early revision risk for younger females (< 50 years) with HRA—more than 7 times when compared to cemented THA—suggesting that HRA is unsuitable for this patient group. This observation is based on 221 HRA cases with 13 revisions, which is a limitation.

### The hip resurfacing group

In previous registry reports, the cumulative total revision rates for HRA after 1 year varied between 1.6% and 1.9% (NJR England and Wales 2007, AOANJRR 2008) and after 3 years they varied between 2.6% and 4.5% (AOANJRR 2008, NJR England and Wales 2009). In the Australian report, the cumulative total revision rate reached 3.7% after 5 years (AOANJRR 2008). The 2-year result for HRA in the NARA database, with a cumulative 2-year revision rate of 3.7% including septic revisions, is close to the high range of these figures.

The Australian, British, and Swedish registries have reported approximately twice the revision rate for women than for men (AOANJRR 2008, Kärrholm et al. 2008, NJR England and Wales 2009). To date, the Australian registry has had the largest number of registered hip resurfacings, and a recent analysis of 12,093 HRAs with up to 8 years of follow-up has revealed an increased risk of revision with increasing age, with smaller femoral head size, with a primary diagnosis of developmental dysplasia, and for certain designs of prosthesis. On adjusting for femoral head size, female sex no longer remained as independent risk factor (Prosser et al. 2010). A similar relationship between sex and femoral head diameter has been reported from a single institution, with 655 BHRs at an average of 3.5 years follow-up (McBryde et al. 2010).

In our analysis of HRA, we found that the revision rate for females was approximately doubled. Further studies concerning different reasons for revision were not done due to the relatively small number of revisions. In addition, femoral head diameter alone had no certain influence on early revision rate but our results suggest that there may be an association between sex and femoral head diameter, which could perhaps be clarified further with more cases and longer follow-up. We could not find any reduced revision risk with increased femoral head diameter or any increased risk with age or with secondary osteoarthritis. Our analysis did, however, include fewer patients and involved a shorter follow-up than the Australian study.

We arbitrarily used a limit of 70 HRAs to separate low-volume hospitals from high-volume hospitals. This limit was set so that all 3 countries should contribute with at least one hospital. This parameter should preferably be split up further to volume per surgeon, but such information is not available in the NARA database. Nonetheless, high volume according to our definition turned out to have an impact on the results in terms of revision. Based on repeated reports of inferior results of HRA, it can be expected that the frequency of these procedures will decrease. If they will still be used in young males, it seems reasonable that the operations would become more centralized in the Scandinavian countries. During the time of collection of data in the NARA database for this study, such operations had already been performed in 48 different hospitals (Denmark: n = 14; Norway: n = 7; Sweden: n = 27).

4 designs could be analyzed separately. The Birmingham Hip Replacement (BHR) device stood out from the others as having a statistically significant better survival, which is consistent with results from the Australian, British, and Swedish registries. 2 of the designs analyzed, ASR and ReCap, had a considerably shorter follow-up than BHR. However, assuming a fairly constant revision rate, this should have amplified the difference measured between BHR and these designs. On the other hand, the results for these 2 designs (ASR and ReCap) may be more likely to have been influenced by early learning curve problems, as described by Marker et al. (2007), Witjes et al. (2009), and Nunley et al. (2010).

Early cup loosening has been reported with the Durom acetabular component (Long et al. 2009). In the USA, this cup has a somewhat different coating with smaller pores than used in Europe. In our study, only 1 of the 16 Durom revisions included exchange of the cup. In all others, only the femoral component was replaced, suggesting that early cup loosening is not a problem with this variation in design.

The reason for the good early performance of the BHR as found by us and others is not known. Also, previous reports have described favorable early results with this implant (Back et al. 2005, Daniel et al. 2004, Treacy et al. 2005). To our knowledge, there has not been any clinical study comparing different HRA designs. There are certain differences between designs regarding metallurgy, articulation clearance, material thickness, implant geometry, and recommended cementation technique but there is limited knowledge of how these properties affect outcome. One cadaveric study has shown that the BHR femoral component has superior cement penetration compared to other designs, including those used in this study (Beaulé et al. 2009), but the importance of this difference is unknown. Apart from metallurgical and design-related factors, other reasons such as instruments used during insertion, educational programs, and procedure frequency per surgeon may have had a positive influence. Such factors are important, but their true influence on the early complication rate is poorly understood, not least for hip resurfacings.

### Conclusion

Hip resurfacing arthroplasty has a higher risk of early revision than conventional and cemented THA. Implant design has an influence on early revision rates, as do hospital procedure volumes. Our 2-year results from the NARA database do not support continued use of hip resurfacing arthroplasty. Further follow-up is necessary to determine whether HRA might still be an alternative in males.
